# Targeting lung cancer stem-like cells with TRAIL gene armed oncolytic adenovirus

**DOI:** 10.1111/jcmm.12397

**Published:** 2015-02-16

**Authors:** Yu Yang, Haineng Xu, Weidan Huang, Miao Ding, Jing Xiao, Dongmei Yang, Huaguang Li, Xin-Yuan Liu, Liang Chu

**Affiliations:** a State Key Laboratory of Cell Biology, Institute of Biochemistry and Cell Biology, Shanghai Institutes for Biological Sciences, Chinese Academy of SciencesShanghai, China; b College of Life Science, Henan Normal UniversityXinxiang, Henan, China; c Xinyuan Institute of Medicine and Biotechnology, Zhejiang Sci-Tech UniversityHangzhou, China

**Keywords:** lung cancer stem-like cells, A549 sphere cells, oncolytic adenovirus, ZD55-TRAIL, apoptosis

## Abstract

Lung cancer stem cell (LCSC) is critical in cancer initiation, progression, drug resistance and relapse. Disadvantages showed in conventional lung cancer therapy probably because of its existence. In this study, lung cancer cell line A549 cells propagated as spheroid bodies (named as A549 sphere cells) in growth factors-defined serum-free medium. A549 sphere cells displayed CSC properties, including chemo-resistance, increased proportion of G0/G1 cells, slower proliferation rate, ability of differentiation and enhanced tumour formation ability *in vivo*. Oncolytic adenovirus ZD55 carrying EGFP gene, ZD55-EGFP, infected A549 sphere cells and inhibited cell growth. Tumour necrosis factor-related apoptosis-inducing ligand (TRAIL) armed oncolytic adenovirus, ZD55-TRAIL, exhibited enhanced cytotoxicity and induced A549 sphere cells apoptosis through mitochondrial pathway. Moreover, small molecules embelin, LY294002 and resveratrol improved the cytotoxicity of ZD55-TRAIL. In the A549 sphere cells xenograft models, ZD55-TRAIL significantly inhibited tumour growth and improved survival status of mice. These results suggested that gene armed oncolytic adenovirus is a potential approach for lung cancer therapy through targeting LCSCs.

## Introduction

Lung cancer is one of the most common cancers in China 1,2 and the leading causes of cancer-related death throughout the world 3. It consists of two major types: non-small cell lung cancer (NSCLC) and small cell lung cancer (SCLC). Although some cases are resectable or responsive to traditional therapy initially, risk of recurrence and drug resistance remains high, leading to a very low 5-year survival rate 4–7.

In the recent years, it was thought that drug resistance, recurrence, initiation and metastasis of cancer cells are resulted from a subpopulation termed as ‘cancer stem cells’ (CSCs). CSCs were first reported in leukaemia 8, and subsequently in solid tumours, such as breast cancer, brain cancer, colon cancer and lung cancer 9–12. Researchers discovered that a rare population in lung cancer cells were able to form colonies in soft agar and tumours in mice about 30 years ago 13. Increasing studies certified the residence of CSCs in lung cancer by using different isolation assays, including cell sorting by certain markers and accumulation in specific medium 14–18.

Since CSCs stay at quiescent status and express high level of ATP-binding cassette 19 transporter family proteins, they exhibit slow turnover and expel anti-tumour drugs 20,21. Conventional treatments that failed to eradicate CSCs might reduce tumour temporarily, however, resistance, relapse and metastasis were likely to occur after treatment suspended. Therefore, it is important to develop an approach capable of targeting CSCs, which is able to eliminate long-term growth of tumour 22.Various types of viruses, such as herpes virus, poxvirus, retrovirus and adenovirus, have been utilized in cancer therapy after modifications, some of which displayed capacity of targeting CSCs 23–26. Adenovirus is widely used in cancer therapy. It is able to infect both non-quiescent and quiescent cells, and would not be expelled out of cells by ABC family proteins compared to chemo-therapy drugs 27. Some work reported the anti-CSCs effect of adenoviruses in oesophageal cancer and breast cancer 28,29, however, its effect in lung CSCs remains unclear. In our previous studies, therapeutic genes armed oncolytic adenovirus (named as Cancer Targeting Gene-Virotherapy) exhibited greater outcome in cancer therapy than oncolytic virotherapy 30–33. In this work, lung cancer stem-like cells (LCSLCs) were accumulated from lung cancer cell line A549 cells which were cultured in serum-free medium. ZD55-EGFP, an oncolytic adenovirus carrying EGFP, was able to infect and elicit cytotoxicity in LCSLCs. TRAIL gene armed oncolytic adenovirus, ZD55-TRAIL, exhibited greater anti-LCSLCs ability than ZD55-EGFP *in vitro* and *in vivo*. This work first demonstrated that gene armed oncolytic adenovirus could infect LCSLCs and suppress their growth.

## Materials and methods

### Cell culture and reagents

The human lung cancer A549 cells were obtained from Cell Bank of Shanghai Institutes for Biological Sciences, Chinese Academy of Sciences (Shanghai, China) and maintained in RPMI 1640 medium supplemented with 10% heat-inactivated foetal bovine serum. All the cells were incubated in a humidified atmosphere with 5% CO_2_ at 37°C.

A549 cells were seeded in ultra-low attachment 6-well dishes (Corning) and maintained in serum-free DMEM/F12 (Hyclone Beijing, China) medium supplemented with 20 ng/mL EGF, bFGF and IGF1 (PeproTech, Rocky Hill, NJ, USA). After adding growth factors (20 ng/ml) for two other days, the propagated spheroid bodies were collected for subsequent experiments. At the same time, A549 cells were cultured in ordinary 6-well dishes with the above medium and growth factor as control.

Adenoviruses ZD55-EGFP and ZD55-TRAIL were previously constructed 31. Embelin and Resveratrol were obtained from Sigma-Aldrich. LY294002 was purchased from Cell Signaling Technology (Beverly, MA, USA).

### Chemo-resistance, cell proliferation and cell cycle analysis

Cell viability was determined by MTT assay to measure cell sensitivity to chemotherapeutics and compare cell proliferation rate. For chemo-resistance, cells were seeded at a density of 5000 cells/well in the 96-well plates, and treated with 5-FU, cisplatin, doxorubicin or vinblastine 12 hrs later. After 48 hrs, cell viability was measured. For proliferation, cell density in the same plate was 3000 cells/well and cell viability was examined at indicated days. In the MTT assay, 10 μl 3-(4,5-dimethylthiazol-2-yl)-2,5-diphenyltetrazolium bromide (MTT, Beyotime Haimen, China) was added into each well and incubated for 4 hrs at 37°C. After removal of the supernatant, cells were dissolved in 100 μl of dimethyl sulfoxide (DMSO, Guanghua Shantou Sci-Tech, China) and the absorbance was measured at dual wavelength of 595 nm and 630 nm by using a Microplate Reader (Thermo Fisher Waltham, MA, USA). The relative cell viability was shown by fold change to the corresponding mock and day 0 for chemo-resistance and cell proliferation respectively. Experiments were replicated for 3 times.

Propidium iodide (PI) staining was used for cell cycle assessment. Cells were collected, washed and suspended in 200 ml PBS, followed by fixation in 70% ethanol overnight at −20°C. Then after wash and incubation with RNase A (20 μg/ml) at 37°C for 30 min., cells were stained with 50 μg/ml PI (Sigma-Aldrich St. Louis, MO, USA) for 30 min. and subjected to Fluorescence-activated cell sorting (FACS) for data acquirement.

### Western blot

Western blot was performed according to the standard procedures. Primary antibodies against XIAP, cIAP1, cIAP2, livin, survivin were purchased from Cell Signaling Technology. E1A, caspase 9, caspase 8, caspase 3, PARP and TRAIL antibodies were obtained from Santa Cruz biotechnology (Santa Cruz, CA, USA), E1B 55KD was obtained from Oncogene (Cambridge, MA, USA) and GAPDH antibodies was from CoWin Bioscience (Beijing, China). All the secondary antibodies were purchased from Santa Cruz biotechnology.

### Quantitative RT-PCR (qRT-PCR) assay

Total RNAs were isolated by Trizol (Invitrogen, Carlsbad, CA, USA) according to the manufacturer's protocol. Reverse transcription was performed with the ReverTra Ace® qPCR RT Kit (Toyobo Osaka, Japan). Expression levels of MRP1, ABCG2, MDR1, Sox2 and Nanog were measured by SYBR® Green Realtime PCR Master Mix (Toyobo) with their specific primers (Table S1). GAPDH was used as a control. Each assay was performed in triplicate.

### Colony formation assay

A549 cells and A549 sphere cells were seeded at a density of 1 × 10^3^ cells/well in 6-well plates. The morphology of colonies was photographed 12 days later. Besides, the colonies were fixed and stained with 2% crystal violet that dissolved in 20% methanol solution.

### Adenoviruses identification

Genome of adenoviruses ZD55-EGFP and ZD55-TRAIL were extracted according to the protocol of Blood Genome Extract Kit (Generay, Shanghai, China). The existence of EGFP or TRAIL gene in the viral genome and contamination of wild-type adenovirus were detected by PCR with corresponding primers (Table S1). The expression of adenovirus replication associated genes and TRAIL gene were examined by western blot.

### Virus infection ability detection

A549 cells and A549 sphere cells were infected with ZD55-EGFP at a MOI of 20 for 48 hrs and analysed by fluorescence microscope and FACS for EGFP positive cells. To measure the cell-entering ability of virus, the genomic DNA was isolated severally from these two types of cells after a 6-hour virus infection at a MOI of 10, according to the protocol of Cellular Genome Extract Kit (Generay). Level of E1A gene was then detected by PCR with GAPDH as a control.

### Hochst 33258 staining

A549 sphere cells were infected with ZD55-EGFP or ZD55-TRAIL at a MOI of 5. 48 hrs later, cells were fixed with 4% PFA (paraformaldehyde) for 15 min., stained with Hoechst 33258 (Molecular Probes, Eugene, OR, USA) at 1 μg/ml for 1 min., and subjected to fluorescence microscope.

### Mitochondrial membrane potential alteration analysis

Mitochondrial membrane potential alteration was detected by using a JC-1 staining kit (Beyotime) according to the manufacturer's protocol. After infected with adenoviruses at a MOI of 5 for 48 hrs, A549 sphere cells were collected, suspended in 500 ml medium and incubated with 500 ml JC-1 working solution away from light at 37°C for 20 min. Then, cells were washed twice and resuspended with staining buffer, followed by FACS analysis.

### Animal experiments

All the animal experiments were approved by the Institutional Animal Care and Use Committee, and performed according to the U.S. Public Health Service Policy on Humane Care and the Use of Laboratory Animals. Four-week old female BALB/c nude mice were purchased from SLAC (Shanghai, China) and raised in the SPF animal facilities of our institute.

To measure tumour formation ability, A549 cells and A549 sphere cells were diluted at gradient concentrations, mixed with Matrigel at 1:1, and subcutaneously injected into the left and right rear back of mice respectively. Total 4 × 10^3^ cells, 4 × 10^4^ cells or 4 × 10^5^ cells were injected into each mouse, and each group includes three mice. The status of tumour occurrence was observed every 4 days.

To observe the anti-tumour effect of adenoviruses on A549 sphere xenografts, 1 × 10^6^ A549 sphere cells were mixed with Matrigel at 2:1 and subcutaneously injected into each mouse. After the tumour volume reached around 90 mm^3^, mice were randomly divided into three groups and intratumourally treated with 100 ml PBS or 5 × 10^8^ PFU viruses (ZD55-EGFP or ZD55-TRAIL) twice at a 1-day interval. The tumour volume were measured every 3 days and calculated as length × width × width/2.

### Statistical analysis

All the data were shown as mean ± SD or mean + SD. Comparison between groups were performed by Student's *t*-test or one-way anova by using R software R is available as Free Software under the terms of the Free Software Foundation's GNU General Public License in source code form. Details can be found at http://www.r-project.org/

## Results

### A549 sphere cells possessed properties of CSCs

**Figure 1:**
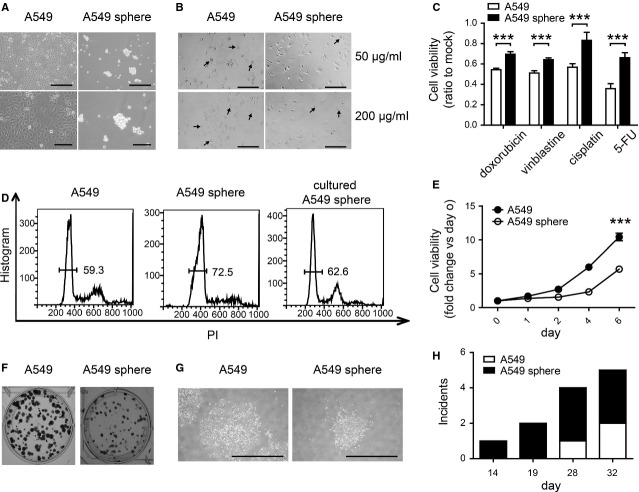
A549 sphere cells have cancer stem cell properties. (A) A549 cells propagated as spheroid bodies in growth factors-defined serum-free medium. Upper scale bar: 500 μm, lower scale bar: 200 μm. (B) Morphological alteration of A549 cells and A549 sphere cells after treated with 5-FU (50 μg/ml or 200 μg/ml) for 48 hrs. Arrows indicate inviable cells; scale bar: 200 μm. (C) Cell viability of A549 cells and A549 sphere cells after treatment with chemo-therapy drugs as measured by MTT assay. The concentration of doxorubicin, vinblastine, cisplatin and 5-FU was 10 μM, 1 μM, 10 μg/ml and 200 μg/ml respectively. (D) Cell cycle distribution of A549 cells, A549 sphere cells and A549 sphere cells cultured for 7 days in medium with serum. Numbers indicate the proportion of G0/G1 phase cells. (E) Cell proliferation rate of A549 cells and A549 sphere cells as measured by MTT assay. (F) Crystal violet staining of cell colonies formed by A549 cells and A549 sphere cells. (G) Morphology of propagated colonies of A549 cells and A549 sphere cells; scale bar: 2 mm. (H) Latency and incidence of xenografts formed by A549 cells and A549 sphere cells. Morphology and staining experiments were repeated 3 times, and representative images were shown in (A), (B), (F) and (G). MTT experiments were repeated 3 times. Data are shown as fold change relative to that of mock-treated cells (C) or cells on day 0 (E). All data shown represent mean ± SD (*n* = 3). ***P < 0.001.

To obtain LCSLCs, lung cancer A549 cells were cultured in growth factor-defined serum-free medium in ordinary or ultra-low detachment 6-well plates as described in materials and methods. A549 cells grew adherently in a single layer in the former condition, named as conditioned A549 cells (Fig. S1A), while forming spheroid bodies in the latter condition, named as A549 sphere cells (Fig.1A). Then some CSC property-related assays were performed on these cells. Flurouracil treatment elicited less morphological alteration in A549 sphere cells compared to A549 cells (Fig.1B). A549 sphere cells were significantly resistant to chemotherapeutic drugs compared to both A549 cells and conditioned A549 cells (Fig.1C and Fig. S1B), suggesting the resistance was not caused by medium change. Since expelling small molecules out of cells and expressing high level of anti-apoptosis proteins are main causes of chemo-resistant, the transcription and expression level of several associated proteins were examined. The mRNA level of ABC transporter family protein MRP1, not MDR1 or ABCG2, increased in A549 sphere cells (Fig. S1C). Inhibitor of apoptosis (IAP) family members were reported to be related with chemo-resistance of CSCs 34, among which XIAP and livin were highly expressed in A549 sphere cells but cIAP1/2 and survivin not (Fig. S1D). Besides, A549 sphere cells exhibited elevated mRNA level of Sox2 and Nanog, underlying its stronger self-renewal ability compared to A549 cells (Fig. S1E). Moreover, the ratio of quiescent G0/G1 phase cells increased in A549 sphere cells and recovered to a similar level as A549 cells after cultured in medium with serum for 7 days (Fig.1D). A549 sphere cells lost their resistance to 5-FU and cisplatin after cultured in medium with serum, predicting their capacity of differentiation (Fig. S1F). A549 sphere cells exhibited slower growth rate and formed smaller colonies as measured by cell proliferation and colony formation assay, respectively, demonstrating their properties of retaining quiescence (Fig.1E–G). Furthermore, *in vivo* tumour formation assay revealed stronger tumour initiation ability of A549 sphere cells (Fig.1H and data not shown). All these results demonstrated that A549 sphere cells have CSC properties.

### Oncolytic adenovirus had cytotoxic activity against A549 sphere cells

**Figure 2:**
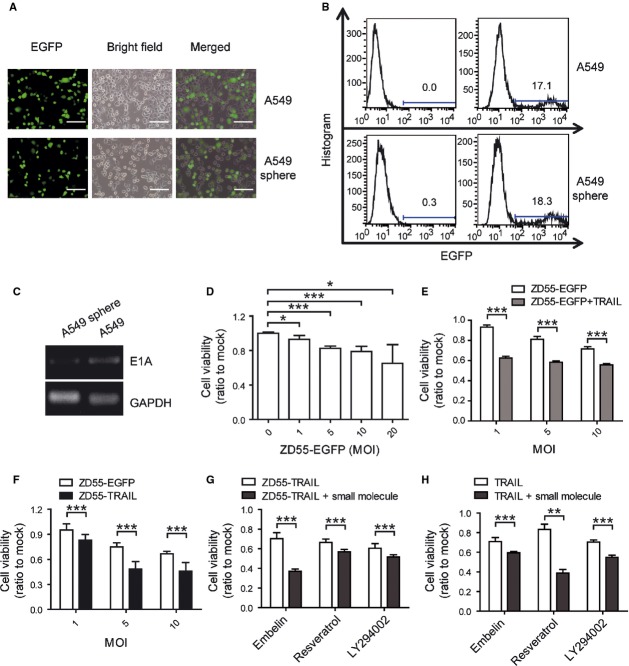
Oncolytic adenovirus infected and had cytotoxicity on A549 sphere cells. (A) Fluorescence microscopy observation of EGFP positive cells after infected with ZD55-EGFP (MOI of 20) for 48 hrs; scale bar: 200 μm. (B) The proportion of EGFP positive cells 48 hrs after ZD55-EGFP (MOI of 20) infection detected by FACS. Numbers indicate the proportion of EGFP positive cells. (C) PCR assay analysis of adenovirus E1A gene in cells after infected with ZD55-EGFP (MOI of 10) for 6 hrs. GAPDH was used as an internal control. (D) Cytotoxic effect of ZD55-EGFP on A549 cells and A549 sphere cells as measured by MTT assay. (E) TRAIL protein enhanced cytotoxicity of ZD55-EGFP on A549 sphere cells as measured by MTT assay. (F) Comparison of cytotoxic effect of ZD55-EGFP and ZD55-TRAIL on A549 sphere cells 48 hrs after infection. (G) Small molecules enhanced cytotoxicity of ZD55-TRAIL. A549 sphere cells were treated with 2 MOI of ZD55-TRAIL combined with or without 50 μM embelin, 50 μM resveratrol or 20 μM LY294002 for 48 hrs. (H) Small molecules enhanced cytotoxicity of TRAIL protein. A549 sphere cells were treated with 100 ng/ml TRAIL protein alone or combined with small molecules for 48 hrs. The concentration of embelin, resveratrol and LY294002 was 50 μM, 50 μM and 10 μM. MTT experiments in (D–H) were repeated 3 times. Data are shown as fold change relative to that of mock-treated cells. All data shown represent mean ± SD (*n* = 3). **P < 0.01, ***P < 0.001. NS, not significant.

A549 cells and A549 sphere cells were infected with the oncolytic adenovirus carrying EGFP gene, ZD55-EGFP (Fig. S2A). Analogous proportion of EGFP positive cells were observed in A549 sphere cells compared to A549 cells (Fig.2A and B). The adenovirus genomic E1A gene was detected in both cells after a 6-hr infection (Fig.2C). Besides, ZD55-EGFP significantly decreased cell growth of A549 sphere cells (Fig.2D). All the data demonstrated that the oncolytic adenovirus ZD55-EGFP can infect A549 sphere cells and suppress cell growth.

To enhance the cytotoxicity of ZD55-EGFP on A549 sphere cells, TRAIL protein was used along with ZD55-EGFP, and the combination treatment significantly decreased A549 sphere cell growth (Fig.2E). Then an oncolytic adenovirus carrying TRAIL gene, ZD55-TRAIL, was applied to infect A549 sphere cells, and significantly inhibited cell growth compared to ZD55-EGFP (Fig.2F, Figs S2 and S3). Small anti-tumour molecules embelin, LY294002 and resveratrol improved the cytotoxic effect of ZD55-TRAIL or TRAIL protein on A549 sphere cells (Fig.2G and H).

### ZD55-TRAIL induced A549 sphere cells apoptosis through mitochondrial pathway

**Figure 3:**
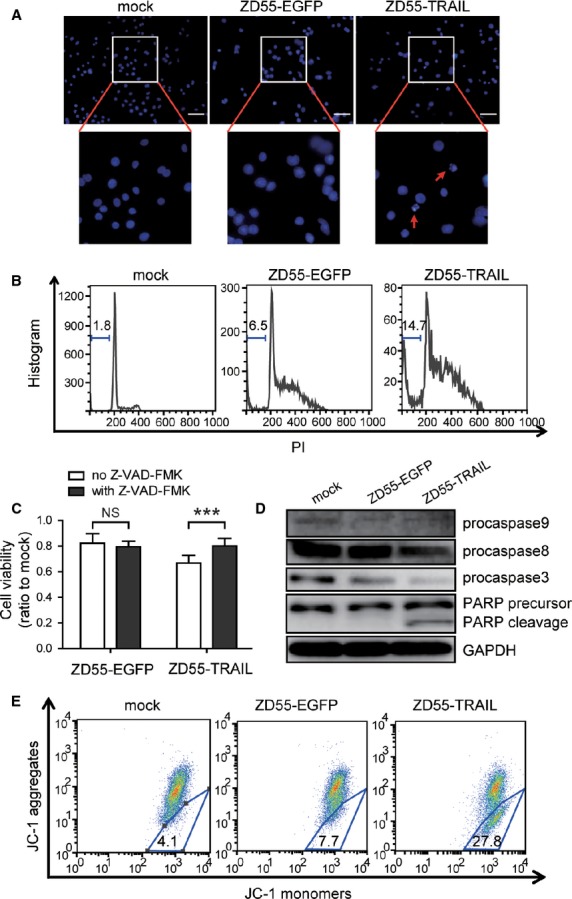
ZD55-TRAIL induced A549 sphere cells apoptosis through mitochondrial pathway. (A) Fluorescence microscopy observation of nucleic fragmentation by Hoechst33258 staining. Arrows indicate fragmented nucleic; scale bar: 100 μm. (B) ZD55-TRAIL increased sub-G1 phase of A549 sphere cells. Numbers indicate the proportion of sub-G1 phase cells. (C) Caspase inhibitor reversed cytotoxicity of ZD55-TRAIL on A549 sphere cells. A549 sphere cells were pre-treated with 10 μM Z-VAD-FMK for 6 hrs and then infected with 2 MOI indicated adenoviruses for 48 hrs followed by MTT assay. Experiments were repeated 3 times. Data are shown as fold change relative to that of mock-treated cells. All data shown represent mean ± SD (*n* = 3). ***P < 0.001. NS, not significant. (D) Western blot analysis of the apoptosis-associated proteins. GAPDH was used as an internal control. (E) ZD55-TRAIL changed mitochondrial membrane potential (Δψ_m_) of A549 sphere cells. Numbers in the trapeziform region indicate the proportion of cells with disrupted Δψ_m_. In A, B, D and E, A549 sphere cells were infected with the indicated adenoviruses at a MOI of 5, and cultured for 48 hrs before further analysis.

A549 sphere cells were infected with adenoviruses for 48 hrs and then went through apoptosis analysis, including Hoechst 33258 staining and PI staining for cell cycle distribution measurement. Proportion of nucleic fragmented and sub-G1 phase cells in A549 sphere cells increased after ZD55-TRAIL treatment (Fig.3A and B). Besides, ZD55-TRAIL induced decrease in pro-caspase 9, pro-caspase 8 and pro-caspase 3 protein level. Caspase inhibitor, Z-VAD-FMK, diminished this decrease, implying that the ZD55-TRAIL-induced A549 sphere cells apoptosis is dependent on caspase (Fig.3C and D). As decrease of pro-caspase 9 correlates with mitochondrial-associated apoptosis, JC-1 staining assay was performed to detect the alteration of mitochondrial membrane potential as described in materials and methods. It revealed that ZD55-TRAIL induced loss of mitochondrial membrane potential in plenty of A549 sphere cells (Fig.3E). These results suggested that ZD55-TRAIL induced A549 sphere cells apoptosis through mitochondrial pathway.

### ZD55-TRAIL inhibited A549 sphere xenograft tumour growth

**Figure 4:**
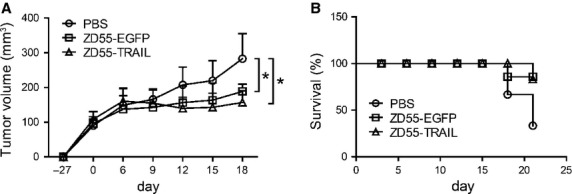
ZD55-TRAIL inhibited A549 sphere xenograft tumours growth. (A) Growth curve of subcutaneous tumours with different treatment. Tumour volume was measured every 3 days. Points indicate mean (*n* = 6), and bars express the meaning of SD. (B) Survival status of mice treated with different adenoviruses; *P < 0.05.

To evaluate the anti-tumour growth capacity of ZD55-TRAIL *in vivo*, subcutaneous xenografts established by A549 sphere cells in nude mice were treated with PBS, ZD55-EGFP or ZD55-TRAIL respectively. Tumours infected with ZD55-TRAIL showed the slowest growth rate compared to the PBS or ZD55-EGFP group (Fig.4A). Moreover, ZD55-TRAIL treatment prolonged the survival rate of the mice (Fig.4B).

## Discussion

Currently, CSCs are isolated mainly by two approaches: cell sorting by surface or functional markers and accumulation *via* specific culture condition. For lung cancer stem cell (LCSC) isolation, CD133 and CD44 were widely used as surface markers 14,15. Functional markers, side population and aldehyde dehydrogenase were also used to isolate LCSCs 16,35. However, there are still some contrary opinions on CSC markers. Expression of CD133 showed no correlation with lung cancer-initiating cells 36. Side population has been disclosed not the CSC in glioblastoma multiforme 37. In this study, lung cancer stem-like A549 sphere cells were accumulated by culturing A549 cells in specific medium, and demonstrated possessing CSC properties, including chemo-resistance, quiescence, differentiation and tumour initiation (Fig.1).

Cancer stem cells exhibit drug resistance mainly because of their capacity of expelling small molecules out of cells or their status of quiescence. Adenovirus possesses its specific manner of infecting CSCs and was unable to be pumped out by the ABC family proteins on the cell surface 27. In this work, oncolytic adenovirus ZD55-EGFP efficiently infected A549 sphere cells and repressed cell growth (Fig.2A–D). With anti-tumour gene TRAIL armed, oncolytic adenovirus ZD55-TRAIL significantly inhibited A549 sphere cell growth *in vitro* and *in vivo* (Figs2E, F and 4). These results suggested that oncolytic virus with therapeutic genes is a potential approach for CSC targeting. Enhanced cytotoxicity of ZD55-TRAIL on A549 sphere cells was achieved by combination with small molecules (Fig.2G and H). It was reported that Emblin, an XIAP inhibitor, enhanced TRAIL-induced apoptosis through posttranscriptional regulation of FLIP 38. LY294002, a PI3K inhibitor, sensitize cells to ZD55-TRAIL-induced apoptosis by suppressing Akt pathway 39. Resveratrol, a natural medicine, elevated the expression of TRAIL receptors and further increased the anti-tumour efficacy of TRAIL.

Recently, many therapeutic targets against CSCs were identified by library screen. Thoridazine, an antipsychotic drug, was disclosed its potent efficiency in eradicating CSCs, and the dopamine receptor that inhibited by thioridazine was correlated with CSCs 40. 6-phosphofructo-2-kinase/fructose-2,6-biphosphatase 4 (PFKFB4) and transformation/transcription domain-associated protein (TRRAP) were elucidated their functions in CSCs through RNAi screen as well 41,42. Adenoviral vector was modified to overexpress or knock down genes in CSCs. For example, adenovirus with shRNA against adenine nucleotide translocator-2 (ANT2) showed its ability of targeting breast CSCs 43. Some self-renewal pathways, such as Hedgehog, Notch and Wnt, play critical roles in CSCs maintenance 44–46. Adenoviruses haboring shRNA targeting these pathways may achieve excellent anti-tumour effect.

Cancer stem cell is an important target in cancer therapy. Oncolytic adenovirus showed its capacity of targeting CSCs, and was considered as a vessel to carry genes or shRNAs to target CSCs, making it an excellent tool for cancer therapy. In the present work, oncolytic adenovirus ZD55-EGFP efficiently infected A549 sphere cells. However, we found that it showed low efficiency in infecting CSCs originated from coxsackie virus and adenovirus receptor (CAR) negative bladder cancer T24 cells (unpublished data). Whereas, fibre-modified oncoltyic adenovirus effectively infected bladder CSCs (unpublished data), indicating that further modifications may be essential in targeting CSCs from different origination. The effect of hTERT promoter modified adenovirus in oesophageal CSCs 28, and several specific promoters modified adenoviruses in breast CSCs have been measured 29,47. Jiang *et al*. constructed an oncolytic adenovirus with modifications of RGD insertion on fibre and 24 base pair deletion on E1A, named as Delta-24-RGD, which presented potent anti-brain tumour stem cells ability 47. These specific elements could be combined in adenovirus modification. Furthermore, combination of two adenoviruses carrying different genes has shown excellent performance in cancer therapy 31,32. Additional works will be required to examine the efficacy of two adenoviruses in targeting CSCs.

## Supporting Information

**Figure S1** Chemo-resistance property of A549 sphere cells and the related gene expression.

**Figure S2** Construction and verification of adenoviruses.

**Figure S3** Cytotoxic effect of ZD55-TRAIL on A549 sphere cells.

**Table S1** Sequences of primers.
