# Supplementary figures and images for: Targeting lung cancer stem-like cells with TRAIL gene armed oncolytic adenovirus
Source: J Cell Mol Med. 2015 Feb 16;19(5):915–23. doi: 10.1111/jcmm.12397 (PMC4420595; doi:10.1111/jcmm.12397)

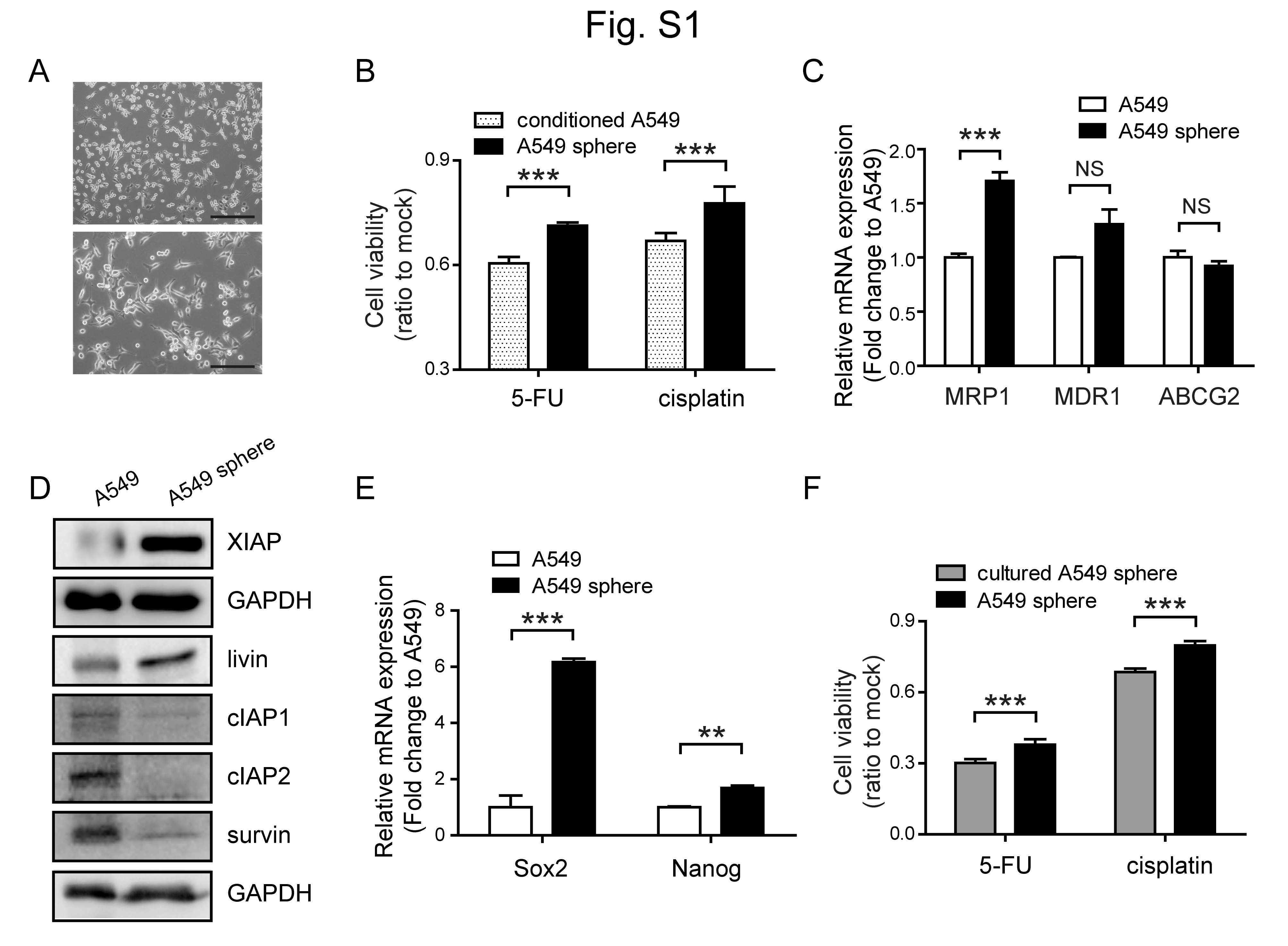

Supplement: Supplementary file 1 [file jcmm0019-0915-sd1.tif]

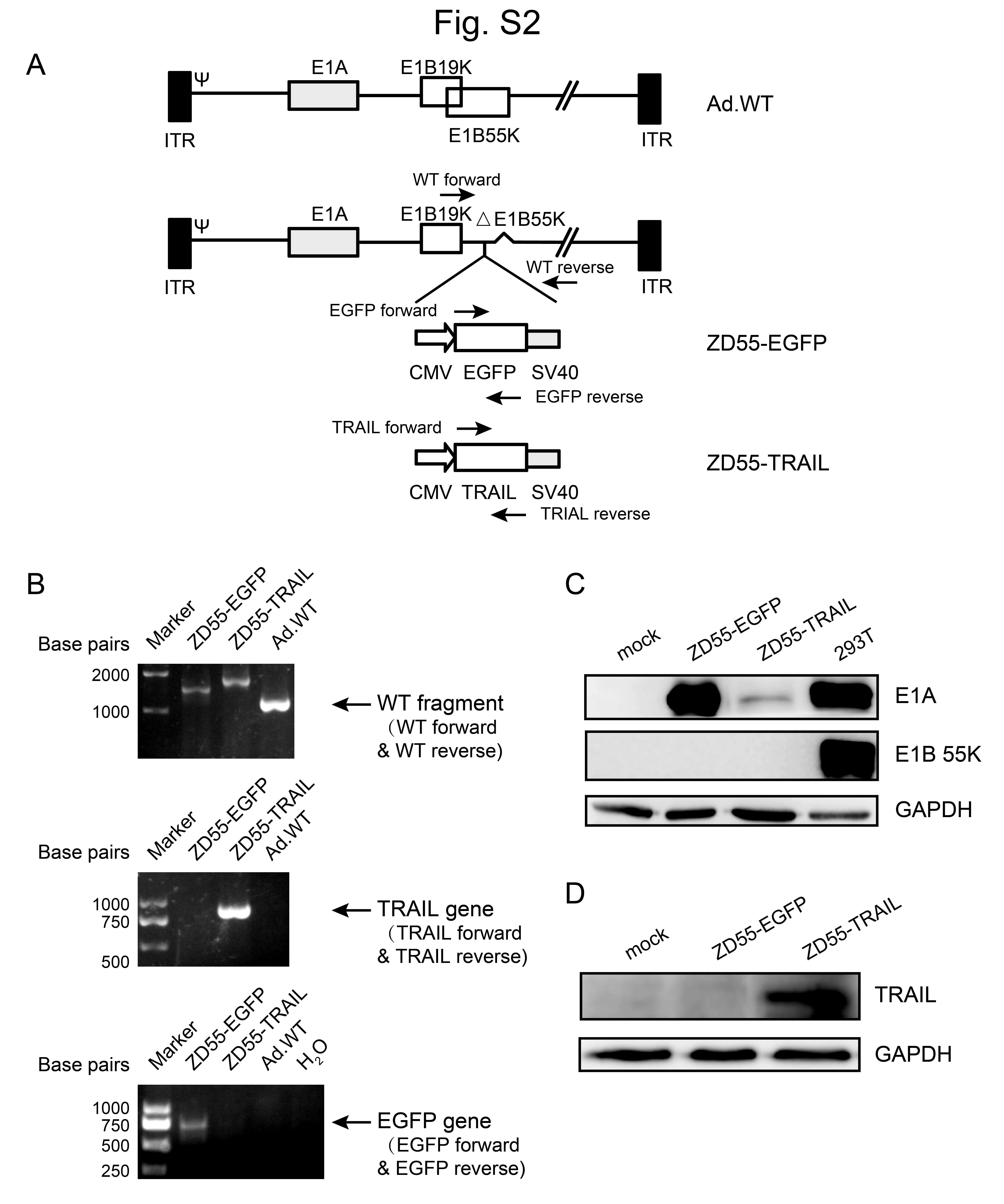

Supplement: Supplementary file 2 [file jcmm0019-0915-sd2.tif]

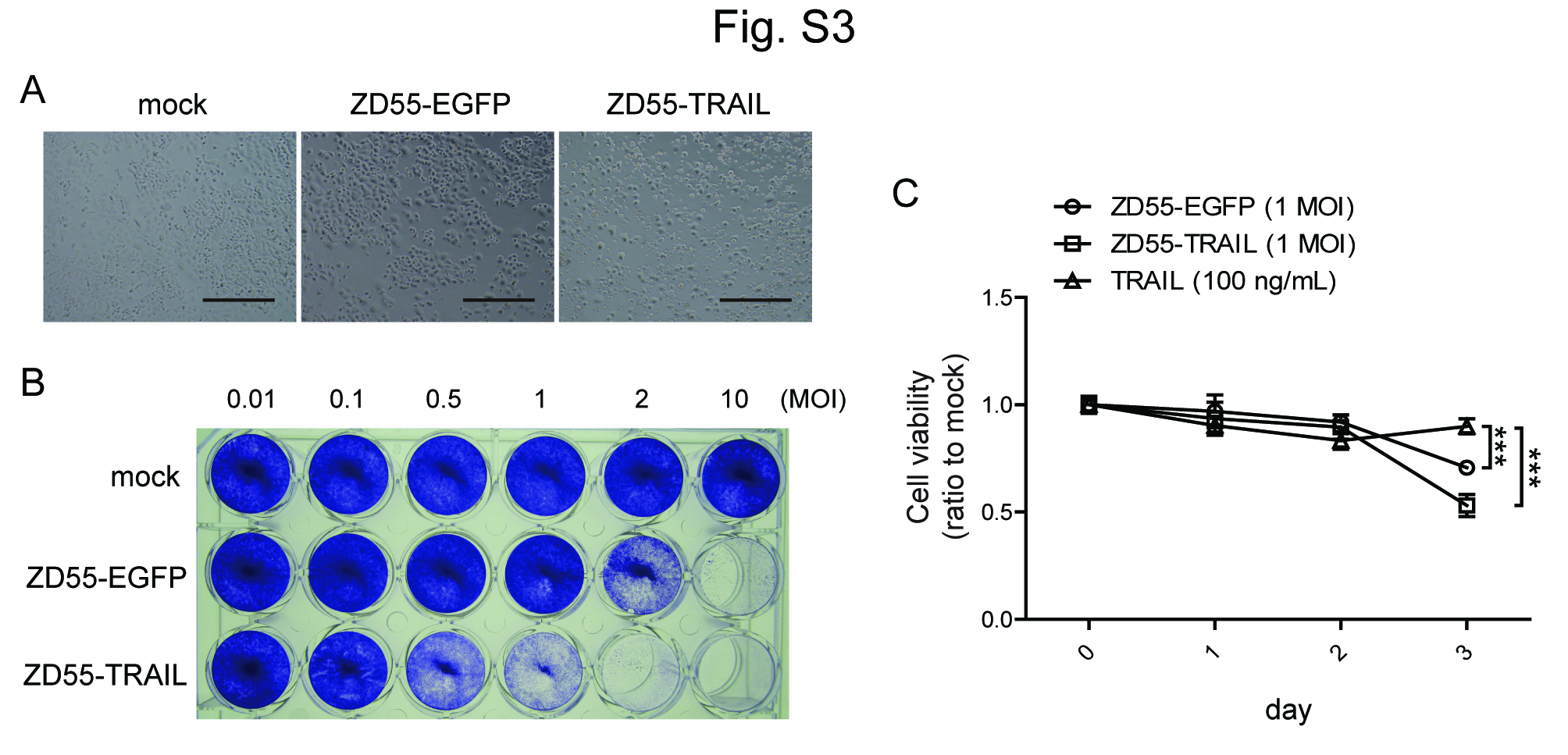

Supplement: Supplementary file 3 [file jcmm0019-0915-sd3.tif]
