# Supplementary material for: Targeting lung cancer stem-like cells with TRAIL gene armed oncolytic adenovirus
Source: J Cell Mol Med. 2015 Feb 16;19(5):915–23. doi: 10.1111/jcmm.12397 (PMC4420595; doi:10.1111/jcmm.12397)
Supplement: Supplementary file 4 [file jcmm0019-0915-sd4.pdf]

Tabs. 1. Sequences of Primers

Primers	Sequences
ABCG2-forward	5’-GTTCTCAGCAGCTCTTCGGCTT-3’
ABCG2-reverse	5’-TCCTCCAGACACACCACGGATA-3’
MDR1-forward	5’-CATTGGTGTGGTGAGTCAG-3’
MDR1-reverse	5’-GTCATAGGCATTGGCTTCC-3’
MRP1-forward	5’-AAGACCAAGACGTATCAGGT-3’
MRP1-reverse	5’-CAATGGTCACGTAGACGGCAA-3’
GAPDH-forward	5’-GTCTCCTCTGACTTCAACAGCG-3’
GAPDH-reverse	5’-ACCACCCTGTTGCTGTAGCCAA-3’
E1A-forward	5’-TTCTCCGGAG CCGCCTCACCTTTC-3’
E1A-reverse	5’-AGGCTCAGGTTCAGACACAG-3’
WT-forward	5’-AGAGCCCATGGAACCCGAGA-3’
WT-reverse	5’-CATCGTACCTCAGCACCTTCCA-3’
EGFP-forward	5’-CTAGAAGCTTATGGTGAGCAAGGGCG-3’
EGFP-reverse	5’-ATCGGGATCCTTACTTGTACAGCTCG-3’
TRAIL-forward	5’-ATGCTTTAAAATGGCTATGATGGAGGT-3’
TRAIL-reverse	5’-ATCGTTTAAATTAGCCAACTAAAAAGG-3’
Sox2-forward	5’-AAATGGGAGGGGTGCAAAAGAGGAG-3’
Sox2-reverse	5’-CAGCTGTCATTTGCTGTGGGTGATG-3’
Nanog-forward	5’-AGAAATCCCTTCCCTCGCCA-3’
Nanog-reverse	5’-TGGTAGAAGAATCAGGGCTG-3’
